# Saccades and pivoting during positive mechanotaxis in larval zebrafish

**DOI:** 10.17912/micropub.biology.001349

**Published:** 2024-10-09

**Authors:** Georgie Davies, Rodrigo De Marco

**Affiliations:** 1 Liverpool John Moores University

## Abstract

Many animals are drawn to prominent sensory cues. Larval zebrafish consistently show attraction to minute water motions (mWMs). This positive mechanotaxis involves scanning-like eye movements (EMs) and changes in body orientation (pivoting), which likely enhance sensory detection. Video analysis shows that EMs increase during mWMs and negatively correlate with locomotion. Both the strength of mWMs and rearing conditions influence EM frequency, with alterations occurring after mWM offset. Additionally, EMs often accompany pivoting. The quantitative data presented here will aid in further exploring the neural bases of visual responses and positive mechanotaxis.

**Figure 1. Saccades and pivoting during positive mechanotaxis in larval zebrafish:**
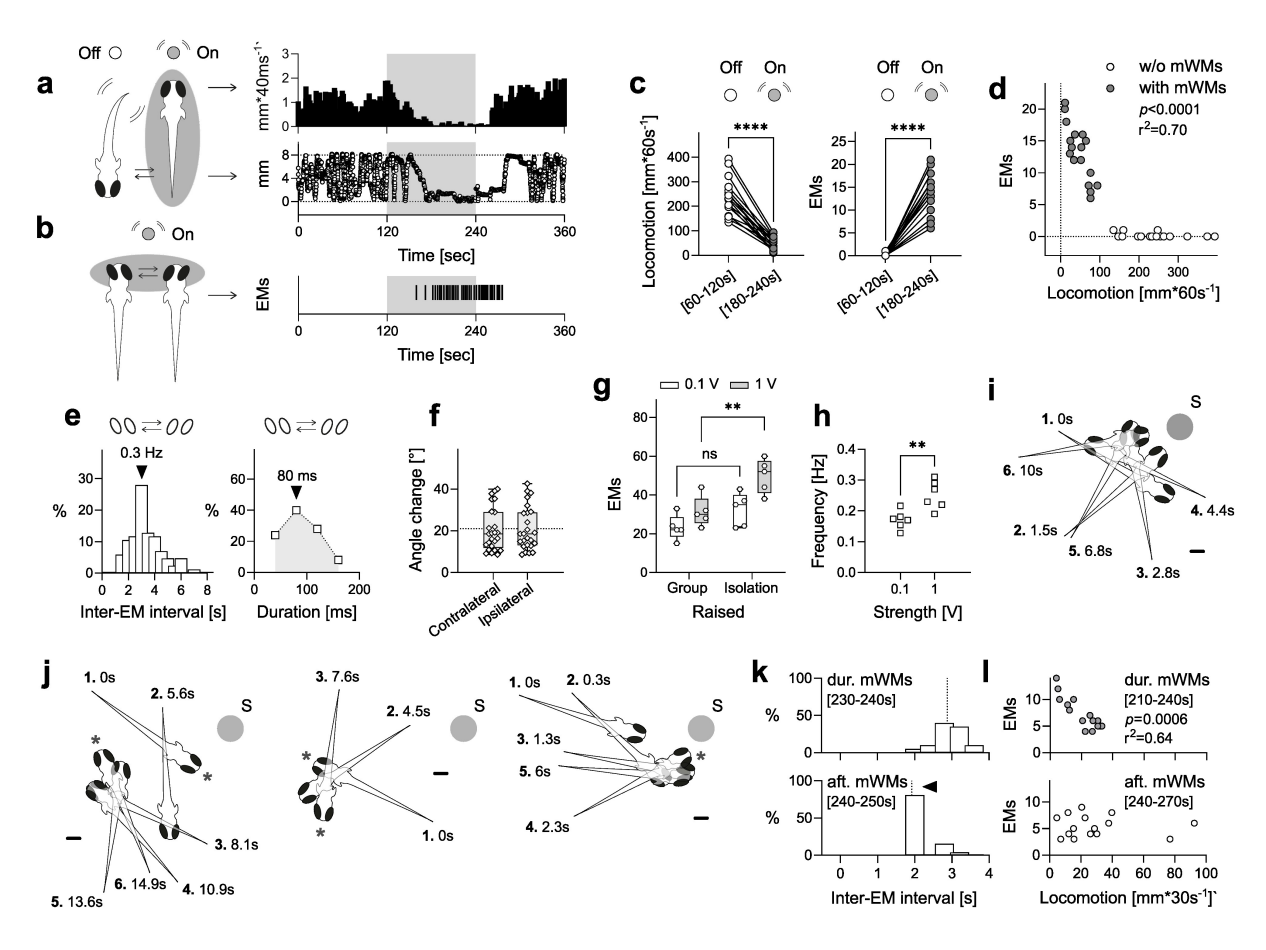
( **a** ) Locomotion level (top) and proximity to the stimulus source (bottom) are shown before, during, and after mWM stimulation. ( **b** ) Slow saccades (side-to-side eye movements, EMs) occur frequently during and immediately after mWM stimulation. The grey-shaded area in ( **a** , **b** ) represents the 2-minute mWM stimulation period. ( **c** ) mWMs significantly reduce motion (left) and increase the frequency of EMs (right) during stimulation. ( **d** ) There is a negative correlation between the number of EMs and locomotor activity during mWM stimulation. ( **e** ) EMs occur synchronously, with a typical frequency of 0.3 Hz (left) and a duration of 80 ms (right). ( **f** ) The angle of EMs during mWM stimulation averages around 20°, with similar changes observed for both contralateral and ipsilateral movements. The dashed line represents the average angle for both groups. ( **g** ) The number of EMs during mWM stimulation increases with mWM strength and is higher in individually raised larvae compared to group-raised larvae. ( **h** ) EM frequency during stimulation depends on the strength of the mWM. ( **i** ) Larvae frequently display pivoting manoeuvres with minimal displacement while approaching the mWM stimulus source. ( **j** ) EMs often coincide with pivoting episodes during mWM stimulation. ( **i** , **j** ) Manoeuvres are numbered in sequence with times indicating their occurrence relative to first movement. Scale bars: 500 μm. Asterisks denote EMs. ( **k** ) The frequency of EMs changes immediately after mWM offset, with shorter intervals between EMs observed right after the offset, followed by longer intervals in the subsequent post-stimulation phase. Dashed lines indicate the medians. ( **l** ) The correlation between locomotion and the number of EMs before mWM offset diminishes after the offset. See Results for statistical details.

## Description

We previously found that freely swimming larval zebrafish ( *Danio rerio* ) are attracted to minute, non-stressful water motions (mWMs) generated locally (Groneberg et al., 2015) . When exposed to mWMs, the larvae gradually reduce their swimming and intermittently move toward the stimulus source, often becoming nearly immobile near the source for the duration of the stimulus. After the mWMs cease, they slowly return to their pre-stimulation levels of locomotion ( Fig. 1 a; see also Figs. 3 and 4 in Groneberg et al., 2015 and Herget et al., 2023, respectively). This overall response, characterized by reduced motion, positive mechanotaxis, and slow recovery of locomotion after the stimulus ends, is highly sensitive to the strength and frequency of the stimulus, as well as to background noise (stationary versus flowing water). While it has been useful for assessing sensory responsiveness and arousal (De Marco et al., 2016; Ryu and De Marco, 2017; Herget et al., 2023) , its full scope remains unclear. Here, we further explore this response to determine whether positive mechanotaxis to mWMs reflects an adaptive search strategy. Specifically, we provide a detailed quantitative analysis of eye movements and changes in body orientation (without significant displacement) that occur during the observed motion damping and movement toward the stimulus source.

Previously, we observed (but did not quantify) that larvae frequently display slow saccades (side-to-side eye movements) and changes in body orientation during periods of reduced locomotion triggered by mWMs. Saccades are also observed immediately afterward, as locomotion gradually increases ( Fig. 1 b). To quantify these saccades and subtle changes in body orientation, we analysed previously recorded 6-minute infrared videos of larvae swimming in complete darkness, with stimulation applied during minutes 3 and 4. We measured the distance swum and the number of side-to-side eye movements (EMs) during the final minute of both the 2-minute pre-mWMs period and the 2-minute mWMs period. The data confirmed that mWMs cause significant motion damping ( Fig. 1 c, left, t(16) = 11.58, p < 0.0001, paired t-test, N = 17) and revealed that EMs occurred frequently during stimulation, whereas they were rare or absent before mWMs ( Fig. 1 c, right, Z = 153, p < 0.0001, Wilcoxon matched-pairs signed rank test, N = 17). Notably, the number of EMs during stimulation negatively correlated with locomotor activity ( Fig. 1 d, grey circles, r(15) = -0.83, p < 0.0001, Pearson’s correlation, N = 17). EMs were synchronous and conjugate, occurring at approximately 0.3 Hz during periods of near-immobility (the histogram of all inter-EM intervals shows a peak at 3 seconds, Fig. 1 e, left), with the most frequent duration being 80 ms ( Fig. 1 e, right) and an average eye movement angle of about 20° from start to end position ( Fig. 1 f, angle change, contralateral: 18.5, 11.8, and 29.05; ipsilateral: 18.5, 13, and 28.85; median and 25th and 75th percentiles, respectively; Z = 45, p = 0.53, Wilcoxon matched-pairs signed rank test, N = 25). These EMs could either be directly modulated by mWMs or occur consistently alongside reduced locomotion, independent of mWMs.

However, three observations suggest that EMs may be modulated by mWMs and play a role in enhancing the detection of relevant stimuli. Firstly, both the level of locomotion and the mean number of eye movements (EMs) closely followed the mWM stimulation protocol. This is evident from the relationship between mWM strength, rearing conditions, and the number of EMs. mWM strength is controlled by the speed of the lateral displacement of a capillary tube submerged in the swimming chamber, which is coupled to a multilayer bender actuator (for detailed technical information, see Groneberg et al., 2015). The input voltage applied to the actuator controls the displacement speed, allowing for precise adjustment of mWM strength. At 0.1V for low strength and 1V for high strength, increased mWM strength results in greater motion damping during stimulation. Furthermore, larvae raised individually—deprived of hydrodynamic stimuli from conspecifics—show a greater response magnitude at the same mWM strength compared to those raised in groups (see Figs. 4E and 6B in Groneberg et al., 2015). The mean number of EMs during stimulation correlates with both mWM strength and rearing conditions: higher mWM strength corresponds to more EMs, and individually raised larvae show more EMs compared to group-raised larvae ( Fig. 1 g, Raised factor: F(1,16) = 14.97, p = 0.0014; Strength factor: F(1,16) = 12.88, p = 0.0025; Raised x Strength interaction: F(1,16) = 1.66, p = 0.22, two-way ANOVA with post hoc comparisons, 0.1V: p = 0.166, 1V: p = 0.0043, N per group = 5). Additionally, the frequency of EMs during stimulation depends on mWM strength ( Fig. 1 h, t(10) = 3.66, p = 0.0044, unpaired t-test, N per group = 6).

Secondly, after the onset of mWMs, larvae approaching the stimulus source while gradually reducing their discontinuous motion often displayed changes in body orientation with minimal displacement, referred to here as pivoting ( Fig. 1 i). In a sample of 25 larvae, 14 (56%) showed pivoting episodes, which were absent before stimulation. These episodes occurred primarily during stimulation, particularly in the second minute, with occasional instances in the seconds following stimulation offset. Pivoting larvae averaged 2.23 episodes (±0.23; range: 1–3, coefficient of variation: 37.3%), each lasting an average of 9 seconds (±1.17 s; range: 1–23, coefficient of variation: 66%). The average cumulative pivoting time was 17.46 seconds (±2.56; range: 2–31, coefficient of variation: 52.78%). EMs frequently coincided with these pivoting episodes (see asterisks in Fig. 1 j), with EMs often occurring between orientation changes when pivoting events lasted longer than a few seconds and involved multiple orientation adjustments. This observation suggests a coordination between EMs and pivoting to enhance sensory detection.

Thirdly, the relationship between locomotion and the number of EMs appears to be sensitive to the offset of mWMs. Although the number of EMs measured over 30 seconds before and after high-strength mWM stimulation did not significantly differ (t(14) = 1.7, p = 0.095, paired t-test, N = 15), the histogram of inter-EM intervals showed a shift in the peak during the first 10 seconds after the mWM offset ( Fig. 1 k). This shift indicates that EMs occurred more frequently immediately following mWMs, with longer intervals between them afterward. Moreover, the mean number of EMs over 30 seconds correlated with the level of locomotion before the mWM offset but not afterward ( Fig. 1 l, r(15) = -0.80, p = 0.0006, Pearson’s correlation, N = 15).

Our previous research found that freely swimming zebrafish larvae respond to minute water motions (mWMs) by reducing locomotion and moving toward the stimulus source, revealing positive mechanotaxis (Groneberg et al., 2015) . This motion damping likely helps minimize self-generated background noise that can interfere with signal detection (Engelmann et al., 2000) . In this study, we observed that during reduced movement, the larvae exhibit scanning-like eye movements with binocular, conjugate saccades, along with pivoting manoeuvres involving minimal displacement. These behaviours suggest an active search for the mWM source as part of a strategy to explore sensory novelty. Our findings, combined with this controlled and straightforward assay, provide a foundation for further investigating the neural mechanisms underlying positive mechanotaxis and the visual responses and circuit motifs involved in these eye movements during mWM stimulation.

## Methods

*Overview*

The primary results in this study build on research previously published by Groneberg et al. (2015). This work provides an extended analysis of video recordings from those initial experiments, focusing specifically on quantifying and exploring the dynamics of eye movements and body orientation changes in response to minute water motions. In the original experiments, breeding and maintenance followed standard protocols. Embryos from a wild-type cross of AB and TL strains were reared in E2 medium at 28°C on a 12:12 light/dark cycle. Larvae were housed in Petri dishes and reared either in isolation or in groups of 20. Tests were conducted on 6-day post-fertilization (dpf) larvae between 9:00 and 18:00, with experimental groups randomly distributed throughout the day. All procedures adhered to national animal welfare guidelines and were approved by the local authority. Larvae were observed under infrared (IR) light in a light-proof enclosure. Their movements were recorded by an infrared-sensitive camera at 25 frames per second. Larvae swam individually in a custom cylindrical chamber containing 400 µl of E2 medium, and their movements were tracked using EthoVision XT software (Noldus Information Technology, Wageningen, Netherlands) and algorithms written in MATLAB 2009b (MathWorks, Inc., Natick, MA, USA). The chamber featured two angled channels: one for the entrance of a capillary tube (outer diameter: 350 µm, full length: 25 mm) that generated controlled, unidirectional lateral displacements (LDs) of its submerged end (lasting 1 ms, applied at 1 Hz) to produce micro-flows during stimulation, and another housing a thermocouple to maintain the medium’s temperature at 28°C via a closed-loop control system. Before testing, larvae were allowed 5 minutes to adapt to the chamber conditions. Detailed descriptions of the experimental setup—including the generation of mWMs, rearing conditions, and behavioural measurements—are comprehensively covered in the original publication (Groneberg et al., 2015) , as well as in subsequent studies (De Marco et al., 2016; Ryu and De Marco, 2017; Herget et al., 2023) . The present study re-examines these video recordings to further investigate the role of eye movements and pivoting during positive mechanotaxis.

*Quantifying locomotion, eye movements, and changes body orientation*

In this analysis, we quantified specific behavioural responses that had been previously observed but not measured in detail. Each infrared video recording featured a single 6 dpf larva and lasted 6 minutes, with mWMs of varying strength (0.1V or 1V) applied during minutes 3 and 4 (for detailed protocols, see Groneberg et al., 2015). Recordings were conducted in complete darkness to isolate mechanosensory input and eliminate confounding sensory modalities. Importantly, zebrafish larvae exhibit eye movements independently of visual stimuli, as these movements are driven by motor commands that do not require light for initiation. We analysed the frequency and timing of eye movements (EMs) and pivoting manoeuvres throughout the entire observation period, which included pre-stimulation, stimulation, and post-stimulation phases. Locomotion estimates were obtained through video-tracking the movements of individually swimming larvae, as described in Groneberg et al. (2015). To quantify eye movements and body orientation changes, video recordings were manually analysed frame by frame using VirtualDub (v1.10.4; Avery Lee, open-source software, www.virtualdub.org ). For each eye movement, the first and last frames showing positional changes were noted, and the duration of the movement was calculated by counting the frames between these two points. Intervals between successive eye movements were measured in the same way. A subset of 25 distinct eye movements, characterized by significant positional changes and a well-defined oval eye shape, was selected for angle and directionality analysis. The angle of movement was measured in Adobe Photoshop (CC 2024; Adobe Inc., San Jose, CA, USA) using screenshots of the first and last frames. Eye movement directionality was classified as ipsilateral or contralateral based on the shift of the upper eye tip. Body orientation changes, referred to as pivoting manoeuvres, were analysed similarly, tracking longitudinal axis shifts where the larva rotated with minimal displacement. The extent and frequency of these pivoting movements were quantified in relation to mWM stimulation and concurrent eye movements.

*Statistical analysis*

Measurements were performed on individual larvae, with each replicate treated independently. Data were assessed for normality and homoscedasticity using the Shapiro-Wilk and KS normality tests, and the Brown-Forsythe and Bartlett’s tests, respectively. For pairwise comparisons involving non-normally distributed data, we used the Wilcoxon matched-pairs signed rank test. Normally distributed datasets were analysed using paired or unpaired two-tailed Student’s t-tests. For multiple group comparisons, we applied two-way ANOVA followed by post hoc tests. Pearson’s correlations were also conducted. All data points, including means or medians (based on distribution), are reported, with no exclusions. Statistical analyses were conducted using MS-Excel (Microsoft Corp., Redmond, WA, USA) and Prism 10.2.0 (GraphPad Software Inc., San Diego, CA, USA).

*Artificial intelligence*

AI or other machine learning tools have not been used in generating any text or images in this manuscript.
